# Fear of Cancer Recurrence as Reminder About Death: Lived Experiences of Cancer Survivors’ Spouses

**DOI:** 10.1177/00302228221123152

**Published:** 2022-08-22

**Authors:** Eglė Urbutienė, Rūta Pukinskaitė

**Affiliations:** 1 Institute of Psychology, 227926 Mykolas Romeris University, Vilnius, Lithuania

**Keywords:** cancer, fear of cancer recurrence, death, spouses, qualitative

## Abstract

Fear of cancer recurrence is the most prevalent and burdensome emotional concern among cancer survivors’ spouses after treatment. This qualitative study aimed to reveal death-related experiences of spouses of cancer survivors in remission, in the context of fear of cancer recurrence. Seven spouses (aged 35–56), four women and three men were explored. Data were obtained using an unstructured interview and analyzed by inductive thematic analysis. The spouses associate cancer relapse with death, risk of losing their spouse. The threat of death triggers not only the feelings of insecurity, uncertainty about the future and loss of control, but also appreciation of life, focus on positive aspects in relationships. Planning for the “worst” scenarios and avoidance helped spouses to reduce tension and enhance control. Interventions for spouses should focus on promotion of emotional expression of death related concerns underlying fear of relapse, also developing new coping strategies to accept and tolerate uncertainty.

Cancer survivorship causes many emotional and physical challenges to the survivors and their families, especially spouses (Girgis & Lambert, 2009). Although the spouses of cancer survivors do not hear the diagnosis by themselves, witnessing the emotional and physical suffering of close one may be traumatic and entails negative consequences. Spouses mostly are the ones, who support the patient, provide assistance with illness management tasks, take additional family and household responsibilities. Furthermore, spouses experience decreased quality of life, psychological distress, fatigue, symptoms of anxiety and depression, post-traumatic stress disorder (De Padova et al., 2021; Mitchell et al., 2013; Posluszny et al., 2015; van de Wal et al., 2017).

One of the most prevalent, persistent and burdensome emotional concerns among cancer survivors’ spouses after treatment completion is fear that cancer may return (Balfe et al., 2016; Simonelli et al., 2017; Smith et al., 2021). Fear of cancer recurrence (FCR) is defined as “Fear, worry or concern relating to the possibility that cancer will come back or progress” (Lebel et al., 2016, p. 3265). The latest systematic mixed studies review revealed that approximately 50% of family members and caregivers experience fear that cancer may return (Smith et al., 2021). Several studies found that this fear was higher in caregivers than in patients (Braun et al., 2021; Simard et al., 2013). Although the literature on caregivers’ fear of relapse has recently grown significantly, yet, no studies have examined, what are the specific content of spouses’ concerns about recurrence, what are the worst fears underlying worries that cancer may come back.

Death is the most common word associated with cancer. It is well recognised that a diagnosis of cancer, a potentially life-threatening illness, is associated with a number of existential concerns such as thoughts about death and dying, confronting one’s mortality and temporality of life (Sharpe et al., 2018). Surprisingly, only a few studies have examined death related concerns in fear of cancer recurrence context, mostly, with patients, not caregivers. Several qualitative studies with cancer survivors have emphasized that the content of concerns about recurrence are often intertwined with fears about death (Cesario et al., 2010; Thewes et al., 2016). Curran et al. (2020) presented theoretical model, where existential factors, such as disruption in core beliefs, death-related concerns, are highlighted in explaining survivors’ fear of relapse. Death anxiety was important predictor of FCR in survivors (Curran et al., 2020; Simonelli et al., 2017). We identified only one study that had examined the association between fear that cancer may come back and death anxiety among cancer caregivers (Braun et al., 2021). Detailed in-depth exploration of spouses’ death related experiences in fear of cancer recurrence context could identify important targets that could broaden knowledge about spouses’ fear of cancer recurrence and improve interventions for fear alleviation.

Therefore, the aim of the study was to disclose lived experiences of fear of cancer recurrence in spouses of cancer survivors in remission. More specifically, this paper focuses on spouses’ death-related concerns in fear of cancer recurrence context. A phenomenological qualitative research strategy was chosen to reveal a complex and little-studied phenomenon of fear of cancer recurrence in spouses and death-related experiences underlying it.

## Method

### Participants

Seven participants (aged 35–56, with a mean age of 45 years), four women and three men were recruited via purposive sampling. This sample size enabled the researcher to become immersed into detailed, in-depth case-by case analysis of each participant’s world, as required by inductive thematic analysis (Braun & Clarke, 2013). The inclusion criteria for purposive sampling were: (1) Married spouses or long-time partners of cancer survivors in cancer remission. However, only married spouses responded to invitation; (2) Time since treatment completion should range from 6 months to 5 years. This period was chosen because during first months after the end of treatment there may be denial of the experience or, conversely, person may be very vulnerable and talking about cancer may be traumatic. A short-term remission period (up to 5 years post treatment) was chosen because survival and mortality rates in oncology are often calculated for a 5-year period. Cancer survivors and their closed ones point out that this period is the most stressful; (3) Voluntarily expressed desire to participate in the interview, because many survivors and their family members, who experience fear of cancer recurrence, try to avoid this topic. No other inclusion criteria were set for the study. Participants differed by survivors‘ cancer diagnosis, received treatment options (see Table 1). Marriage time ranged from 10 to 29 years. Six of the seven participants had more than one child. Participants were recruited through sharing invitation about the study in cancer-related groups on social networks.

**Table 1.** Summary of Participant Details.

Anonymised Name	Age, y	Diagnosis of Cancer Survivor	Treatments Received	Time Since Treatment Completion	Age of Children
Kristina	42	Acute leukemia	Chemotherapy	1 y	3 and 8 years old
Irena	56	Tongue cancer	Surgery, chemotherapy, radiation therapy	3 y	16 and 33 years old
Tomas	52	Breast cancer	Surgery	4 y	28 years old
Donatas	45	Breast cancer	Surgery, chemotherapy	1 y	15, 11 and 6 years old
Lina	35	Spinal cord tumor	Surgery	9 months	3 and 5 years old
Marija	46	Appendix cancer	Surgery, chemotherapy	2 y	21, 15 and 11 years old
Gintaras	39	Breast cancer	Surgery, chemotherapy, radiation therapy, biological therapy, immunotherapy	1 y	5 years old

### Materials

Data was collected applying an unstructured individual interview. Open-ended questions were asked to reveal experiences of fear of cancer recurrence. Interviews began with an open-ended question that allowed participants to choose when and how to speak about fear of recurrence: “Tell me what thoughts you had after reading the invitation to share your experiences about fear of cancer recurrence”. An interview guide was prepared in advance to explore when and how spouses experience fear of cancer coming back, what would be the worst if cancer comes back. While talking about worst fears underlying fear of recurrence death-related concerns arose from participants. The following questions aimed to clarify and expand the content of these concerns – i.e., “How do you feel when you think you may lose your spouse?”, “Tell me more about this…”. The main task for interviewer was to follow participants and be responsive to their answers, to encourage spouses to provide in-depth, detailed information.

### Procedure

Ethical approval was obtained from the relevant institution. Face-to-face interviews (∼50–120 min in length) were held in 2020. Interviews were conducted by the clinical psychologist, working in the field of psycho-oncology. The interviewer did not have any previous relationship with participants. Locations of interviews were held in NGO Centre of Psychosocial Oncology. Informed written consent was obtained from all participants involved in the study. Interviews were audio taped. Strict confidentiality and anonymity requirements were observed throughout the research in order to protect participants’ personal information. Due to the sensitive content of the interview, participants were provided with information about free psychological help. Audio files were transcribed verbatim.

### Data Analysis

Death-related concerns in fear of cancer recurrence context is complex, little-studied phenomenon, so inductive thematic analysis was selected as appropriate method for identifying, analysing and describing general patterns within data. This method also involves interpretation in the processes of explaining data more in-depth (Braun & Clarke, 2013). Inductive thematic analysis allows to analyze data in semantic (what participants actually express in words) and latent level (what is said “between the lines”, non-verbal language, repetitions). So hidden, avoided and silenced aspects of phenomenon is researched. Thematic analysis is unique as it provides clear data analysis procedure to follow*,* but can be applied within different theoretical paradigms and epistemiological positions. The researcher took personally and professionally close phenomenological epistemiological orientation, which emphasizes on subjective, unique human experience, relies on participant as expert of their own condition.

During analysis themes were identified in a data-driven inductive way. Developing a saturated phenomenon description was sought. According to Braun and Clarke (2013), analysis process was conducted in the following steps. (1) The researcher immersed themselves in the data through transcribing it, multiple readings of transcript and noting initial ideas in the researcher’s diary. (2) Further, initial codes were generated through open inductive coding. This phase involves the production of semantic (i.e., “Express worry about their own health”) and latent codes (i.e., “Uncontrollability of death provokes anger”) codes. All the relevant features of the data were coded across the entire data set. (3) Recurring initial codes were collated into potential thematic units, e.g. the theme „Heightened sense of insecurity“ is consisted of codes such as “Living with sword of Damocles”, “Cancer is ongoing, threatening, never ending event”, “Loss of control”, “You are sort of suspended in the air”. (4) The analysis continued by reviewing primary themes, checking how themes relate to the coded extracts, defining and naming themes. (5) While producing the report, clear, vivid, and the most substantive quotations were selected for the illustration of the analysis. During analysis process, the researcher participated in individual and group supervisions with other qualitative researchers to increase research credibility and transparency of the researchers’ decisions, interpretations.

## Results

The research findings have revealed that the spouses of cancer survivors in remission experience fear that the disease may return. Spouses experience fear of cancer recurrence as reminder about death. The research participants have indicated the events and situations that trigger thoughts about potential loss of a spouse. The threat of death evokes the feelings of insecurity, uncertainty, and loss of control in them. Moreover, the spouses have discussed what helps them to reduce the tension caused by the threat of death.

Based on the data, the following four themes have been generated to reveal death-related experiences of spouses of cancer survivors in remission, in the context of fear of cancer recurrence (see Table 2).

**Table 2.** Themes of Death-Related Experiences of Spouses of Cancer Survivors in Remission.

Themes
1. Situations that trigger the fear of cancer recurrence and the threat of death
2. The worst fear is the loss of the loved one
3. Heightened sense of insecurity
4. The defeat of death with life

### Situations That Trigger the Fear of Cancer Recurrence and the Threat of Death

This theme focuses on the situations and events where the participants undergo the fear of cancer recurrence as well as the threat of death of their spouse.

The participants associate the physical symptoms experienced by their spouse (e.g., pain) with cancer recurrence: *“Existent non-existent pain immediately evoked the thoughts about metastases.”* (Tomas); *“Any allergy made you think about possible recurrence.”* (Gintaras). Lina tells that tension is further escalated by her husband’s exaggerated attention to his physical sensations, especially after his friend’s death of stomach cancer: *“When he has his stomach examined, it’s always like an allusion to his friend… and he always, ‘what if it’s cancer.’”*

Patient visits to doctors constitute a direct encounter with the fear of cancer recurrence for the spouses. Waiting for health examination results also provokes thoughts about the worst possible scenarios, e.g., cancer recurrence, death. Irena confesses that when going for medical check-ups with her husband, they both experience physically a lot of tension and anxiety before the examination and while waiting for the results. Good medical test results are like a permission to live again for her husband and for her: *“When we learn that everything is okay, then life starts anew and we run, and we dream, and we think. But before that – really profound emotions, your heart feels so heavy. There is this excruciating pain in the chest… I don’t even want to talk about this”.* Kristina remembers the situation when the thoughts of cancer recurrence were evoked by an unplanned visit appointed by her husband’s doctor following a regular check-up: *“On Saturday evening, he received a message about a visit to a haematologist. It had never happened before; why was I invited? We ran through all possible scenarios in a single day. <…> It’s terrible (inhales). You switch to a different mode. Stand-by mode, tension mode. You start analysing, this never happened before. Why am I going there?”.*

The information available from doctors, scientific articles on unfavourable cancer prognosis, the risk of recurrence also provoke thoughts of recurrent cancer and death in the research participants: *“We were told that everything was clear. We shouldn’t expect anything. <…> If at least one doctor told me that everything would be fine, that the cancer wouldn’t come back, it would be totally different… but now you are just bombarded with the information that it will.”* (Gintaras); *“I no longer recommend reading any statistics or average terms online. In my husband’s case, his cancer was mucinous, the worst possible cancer. The prognosis is really grave (sighs).”* (Marija). One of the research participants admits that he associates recurrent cancer with limited treatment options and sudden death. Circulating stories about other patients who died quickly due to cancer recurrence get engraved in memory. Anxiety is further escalated by uncontrolled course of the disease: *“Why this fear of cancer recurrence is so big, <…> oftentimes treatment has to be changed and every subsequent treatment applied is less effective. <…> alongside the treatments, this can simply mean a sudden death. There are such cases. When cancer recurrence is sudden; and Rūta’s disease was really aggressive.”* (Gintaras).

The loss of relatives and friends who had cancer strengthens the threat of a spouse’s death. The feeling of self-identification arises with those deaths that occurred at approximately the same time as the diagnosis of cancer of a spouse: *“My mother’s death (sighs, pauses), you do project and you think that now my mother has died, but there is one more threat; that presence of death is so obvious.”* (Marija); *“There was this terrible event – we buried our friend in the summer; also 37 years old, and he left a small baby behind. He told us about his stomach cancer in the week between Christmas and the New Year and we buried him in August. It was a terrible blow for him. For me too, when I saw this little girl, I immediately had an association with my fear.”* (Lina). One of the research participants says that her husband’s illness stirred her childhood emotions when her father had cancer. Her spouse’s cancer is like a repeated childhood trauma – father’s death of cancer and her participation in the process of dying – accompanied by the feelings of fear, helplessness, and guilt: *“It was the second time that I had to live through this experience, my father had died of cancer. I was so afraid of my dad. During his funeral, I couldn’t even stand at his coffin, I was so scared. I felt guilty that I hadn’t been able to help him, to talk to him, but I was so scared.”* (Irena).

In summary, visits to doctors, physical symptoms of a patient, unfavourable cancer prognosis imprinted in memory, and deaths of relatives of cancer evoke a persistent feeling of threat that cancer may return at any time and a spouse may die.

### The Worst Fear is the Loss of the Loved One

Spouses of cancer survivors usually associate cancer recurrence with death, with the threat of losing one’s loved one. They refer to this as the scariest and most painful experience behind the fear of cancer recurrence. The present theme reveals the experiences related to the risk of losing one’s spouse and that significant relationship.

Most participants confess that the most dreadful thing would be to lose their husband or wife. The death of a spouse is associated with a psychological crisis, the loss of an important part of one’s identity. The emotional bond where you are valued and loved is lost: *“I think I’d be plunged into a state of profound crisis. <…> As a personality. This would be horrible (tears up). I’m so afraid. <…> I can’t imagine my life without him.”* (Irena); *“That person is so dear to you. We’ve lived together for so long and I feel that love, and I love. And I’m terrified even to think. It’s not a blood relation but rather a really strong emotional connection. The connection of trust, love. And I sometimes think who else if not Petras would care for me more.”* (Marija).

A spouse’s death is regarded as the collapse of the vision of common life and future, safe and planned future, dreams. The death threat posed by cancer is like premature, untimely separation causing the feelings of injustice and anger: *“And that realisation that (tears up) for some reason one can be lost, I think it’s just too early; we have been dreaming of growing old together, of how we’d be sitting at a nice white table with lavenders drinking coffee and feeling happy, and all of a sudden, my illusion is shattered. <…> So, death is terribly unfair.”* (Irena). The threat posed by spouse’s cancer recurrence and death triggers the feeling of loneliness. Marija recalls the early death of her father and her mother’s life as a widow; she tells that the most terrible thing after losing a husband is to remain all alone: *“I’m terrified of being left all alone. <…> My mom lived alone; my dad died really early. And my mother lived alone for 20 years. And I saw how difficult this was. So, perhaps that fear of death is this loneliness.”*

When reflecting on the significance of the loss of their spouse, the participants with small children say that it would be most unbearable to see their children suffering in the face their mother’s or father’s death. The desire to protect a child from grief after possible loss causes tension, sadness, and helplessness realising that it is impossible to protect others from painful experiences. The participants accept the loss of one of the parents in the childhood as the shattering of child’s happiness, and the loss of full-fledged life in a full family: *“To see the children, well, they would suffer anyway… Perhaps that would be most horrific, saddest. The kids would see their helpless mom or would lose her completely (inhales).”* (Donatas); *“What breaks my heart is the children; if something is wrong with my husband, and the kids are still so small and they would lose so much. For the children, it’s like taking away a part of their world. I want them to have a healthy father, and I imagine that their life would be of better quality, they would be happier.”* (Lina). Gintaras experiences a frustrating feeling of guilt over his child, committing himself deeply to saving the mother for his child in this struggle with cancer, death: *“I’m terrified to look Kajus in the eye, I’m feeling guilt (long pause) that I may be unable to save his mother… That I may disappoint them.”*

To one of the participants the threat of loss of her spouse has triggered the fear of being left alone in case she got cancer. Speaking about her fear she laughs to suppress the tension: *“There are lots of stories when people are left when they become ill, well, it has never occurred to me to leave him. <…> I just sometimes have questions whether I would be loved like this, whether he would stay with me (laughs). If I got cancer (laughs).”* (Marija).

The findings reveal subjective experiences of the risk of losing the loved ones. Even though the participants share their thoughts on this sensitive topic of possible loss of their spouse relatively openly during the interview, their non-verbal language (sighs, longer pauses, the use of the second person “you” when speaking, smiles, laughter) reveal tremendous efforts to contain their feelings, to suppress their anguish, tears, or the desire to step back, distance themselves from the theme of death.

### Heightened Sense of Insecurity

The present theme focuses on the feelings of insecurity, uncertainty, and loss of control experienced by the spouses in the face of the threat of death.

Even when the treatment of the spouse’s cancer has been completed and remission has been achieved, the unpredictable course of the disease triggers the feeling of insecurity in the participants. Cancer is accepted as a continuous, threatening, ongoing event. Marija confesses that the risk of cancer recurrence urges her to stay alert and prevents her from returning to her usual rhythm of life: *“I’m feeling that sword of Damocles; the cancer is in remission, but I’m afraid of even allowing myself to think that cancer is retreating. I’m afraid that if I start thinking that everything is okay and we have finally returned to normal life, it will hit again.”*

According to the participants, the likelihood of cancer recurrence and the threat of death evoke the feelings of uncertainty about the future and loss of control. Seeing a spouse in constant cancer follow-up care heightens that troublesome state of uncertainty: *“Uncertainty and that constant suspension in the air. You’re sort of suspended. You’re constantly under follow-up care. And everyone speaks about this; that the disease can return, that you have to monitor yourself.”* (Gintaras). According to Donatas, the threat of cancer and death trigger anxiety and anger because there is nothing you can change:*“Looking into the future, that notional waiting for cancer, death; that life in constant waiting is slightly unnerving.”*

Several research participants have also expressed a heightened feeling of insecurity about their own health and life: *“When my mother got cancer, and then my husband, it’s natural that this fear tends to resurface from time to time; that I haven’t had an ultrasound performed, I haven’t swallowed a tube. That feeling is always with you somewhere behind.”* (Marija). In the face of cancer of a close person, the dividing line between a sick and healthy person disappears. The threat of death is brought closer to a spouse:*“And I think that we all walk along this very narrow line; we all do (inhales).”* (Irena).

The interviews with the participants have revealed that the feelings of uncertainty and loss of control are also caused by confusion – how to stay close and offer help when a spouse is undergoing the fear of cancer recurrence. When one becomes a witness of intensive and unpredictable emotions, there is this strong desire to solve the situation in a rational manner, to control the other’s feelings and yet all such efforts are ineffective: *“Those anxiety attacks… Indeed, there was uncertainty as to what to do because (exhales) it was obvious that logical arguments weren’t working, and the effect of emotional arguments were short-term. It’s obvious that the person is going through a rough patch, but you just don’t know what to do.”* (Tomas);*“Well, probably the hardest thing is to listen, to hear all those fears, all that exaggerated reaction to physical sensations, because there were multiple times when I had to call an ambulance.”* (Lina).

### The Defeat of Death With Life

This theme encompasses the coping techniques employed by the participants that help reduce the threat of death. The appreciation of life, focus on positive aspects of the relationship with a spouse, religiosity, planning and control, minimisation of significance of death, avoidance are just some examples of the efforts to alleviate the feeling of insecurity and the tension posed by the threat of death.

According to the participants, the perception of the limitations of life and its temporality activates that engine of life and encourages them to use the time at hand to the fullest, to enjoy life “here and now,” to appreciate life: *“Life is very short and we have to have as many arrangements, beautiful moments as possible. To go somewhere. To visit someone. To stay together. <…> The fear about his health has been really exhausting, so the ability to enjoy the present moment is very important.”* (Irena); *“Nobody knows how much life has been given to them. A lot of things happen all over the world. Now we have this corona virus. People are dropping like flies. And you never know. It’s important to have a quality life. To live in this day. To enjoy and just to be happy. Not to think about what’s going to happen tomorrow. You just can’t predict everything.”* (Kristina).

According to Marija, the feeling of temporality and the threat of death caused by cancer has actually brought about positive changes in her relationship with the husband, greater appreciation and acceptance of the other without any prior expectations: *“We demand less from each other. <…> I no longer have any expectations as to what he should do as a man, but he doesn’t want this. Our life is temporary, so what can you possibly do.”*

One of the research participants has also indicated religiosity as a cornerstone of security in this insecure, uncertain situation: *“I’m religious (inhales deeply). This is my foundation. My faith and realisation that there is someone above us with whom I can connect and who can help me. You actually understand that you are not alone. There is help, for sure.”* (Kristina).

Another way to reduce tension that the spouses have shared is planning and control in the face of threat. According to Gintaras, the knowledge and information about cancer and the latest cancer treatment methods help him feel in control of the situation. Security is further enhanced by having a ready action plan in case of cancer recurrence: *“We have certain knowledge and precautions (pauses, inhales) for cases, ‘what if,’ what if there’s cancer recurrence and progression; we know what we will do.”* Kristina feels in control of the situation through her focus on health behaviour, i.e., physical activity, strengthening the immune system, nutritional care, and concern for emotional health: *“I do my best to promote health together with my family. To strengthen my immune system, to refuse certain bad things, to try not to be angry, not to hold bad emotions. To try to help myself in all possible ways to function well.”*

To lessen the fear of death and the tension regarding potential loss of a spouse the research participants seek to minimise the power and significance of death. In the opinion of the participant Irena, death is not the end but rather a different state that we transit into: *“I think I’ve reached such a level (a short pause) when death is no longer so important. I’ve now accepted death as a different state that doesn’t have to be given such prominence.”* When speaking about death as a natural, inevitable fact of life, Tomas recalls the book he once read that concentrated on the suffering caused by immortality: *“The main point of the book is that at the dawn of the 20*^*th*^ *century, Rubashov the gambler challenges the Devil to a game of cards, loses the game and is granted immortality as a punishment. And the whole story line develops around his torments regarding this immortality (laughs).”*

The participants have also admitted that sometimes they try to deliberately escape their emotions and the tension provoked by the theme of death: through engagement in active sports activities and increased workload (*“I try to get away from that anxiety. I start working even harder. I do a lot of sport, I cycle a lot, and then it all gets sorted out. <…> I remember when Jonas was being operated on, I was cycling; even when my mother died, I cycled.”,* Marija); with the help of laughter and irony (*“When she said that she would die, I looked at that with a certain shade of irony. <…> How do you know? Maybe I’ll be the first to go.“,* Tomas); by avoiding any thoughts about cancer recurrence or death (*“There’s this kind of block, and I don’t ponder on this too much, that it could be really bad. It’s like some procrastination. It’s easier like this now, otherwise it would be too hard.”,* Lina).

According to the participants, speaking to their spouse or other relatives about the possibility of cancer recurrence or the threat of death only triggers greater anxiety; furthermore, they do not want to be a burden to others: *“We do talk with my husband. Only this heightens his anxiety. If I’m worried, that worry passes on to him (inhales). So, I choose to cycle instead and not to speak about this, because what can you possibly say?” (*Marija); *“I have people I can trust and talk to but I just don’t want to burden them with my troubles.” (*Irena).

In conclusion, even after the completion of cancer treatment and the achievement of cancer remission, the survivors’ spouses experience the fear of cancer recurrence. They associate cancer recurrence with death and risk of losing their spouse. Unpredictable course of the disease and the threat of death trigger the feelings of insecurity, uncertainty about the future, and loss of control. The coping techniques employed by the spouses – appreciation of life, religiosity, planning and control – help them ease the tension and feel more secure in the face of the threat of death.

## Discussion

A qualitative analysis aimed to reveal death-related experiences of spouses of cancer survivors in remission, in the context of fear of cancer recurrence. Findings have disclosed that spouses experience fear that cancer will return as reminder about death. Based on the data, the following four themes have been generated: situations that trigger the fear of cancer recurrence and the threat of death; the worst fear is the loss of the loved one; heightened sense of insecurity; the defeat of death with life.

While talking about their fear of relapse all participants expressed death-related concerns. Even when cancer survivors are in remission, spouses experience illness as ongoing, life-threatening event in the present. Our study disclosed that spouses associate cancer recurrence with death sentence, limited treatment options, aggressive and uncontrolled course of cancer, fast and sudden death. These associations arise from heard negative information from doctors, internet about high risk of recurrence, bad prognosis, also stories of other cancer patients, who died. Such illness representations may emerge due to unpredictable and uncontrolled nature of cancer, also influenced by general ideas and cultural knowledge of cancer in society. Robb et al. (2014) qualitative study revealed that healthy individuals who had never had cancer associate cancer with trauma, fear and death. Furthermore, despite improvement in treatment outcomes, they reported that it is hard to accept that people survive. Systematic review conducted by Vrinten et al. (2017) identified that fear of death was the most common concern what people fear about cancer.

Our study data indicated dynamic and fluctuating nature of experienced threat. Spouses shared that physical symptoms of the survivors, follow-up cancer care visits, waiting for medical examination results increase thoughts about possible cancer recurrence and threat of death of a spouse. Similar external triggers (i.e., medical appointments and tests, survivors’ physical changes) about relapse are indicated by previous studies with caregivers and family members (Smith et al., 2021). Despite successful initial treatment, these situations remind that cancer may return at any time. Unpredictable cancer trajectory generates to spouses the feeling of insecurity and encourages vigilance to survivors’ bodily simptoms. Fardell et al. (2016) noted, that intimidating interpretation of physical symptoms may be due to lack of information about potential signs of cancer relapse. The spouses do not experience physical changes by themselves, they rely on survivors‘ physical condition appraisal. If the spouses do not have information, how to distinguish worrisome symptoms from benign ones, it is easy to misinterpret survivors‘ symptoms and accept them as menacing. Educating spouses about potential signs of recurrence, identifying situations when fear of cancer recurrence and threat of death increases appear beneficial.

Furthermore, our study revealed that the loss of relatives or friends who had cancer evoke a persistent feeling of threat that cancer may return at any time and a spouse may die. The spouses were most affected by those losses where they witnessed the dying process. The death and dying of family members, friends was accompanied by the feelings of fear of disfigured, dying body, helplessness in face of death, guilt for not being able to help a dying person. Certain vulnerability factors (such as past traumatic life events, previous losses of significant others from cancer) may enhance thoughts that cancer may return and reinforce a sense of threat of death (Almeida et al., 2019; Fardell et al., 2016). Thus, it is important to take into account the individual’s trauma history – what past experiences may awake the thoughts about possible cancer recurrence and affect the current sense of threat of death.

The worst fear underlying worry about cancer recurrence was the loss of the spouse. Cancer diagnosis and possibility of cancer relapse caused awareness about their spouses’ mortality. The loss of a spouse was associated with the psychological crisis, the loss of an important part of one’s identity, the collapse of the vision of common life and future. These reflections show the subjective significance and meaning of the relationship with the spouse, also the magnitude of a potential loss. Even the time of death is uncertain and cancer trajectory is unknown, spouses who associates cancer recurrence with death of a loved one may suffer important losses in relationships or collapse of future plans. These findings are in line with previous studies, which revealed that the feeling of loss may exist even before the patient’s death and manifest as relational losses, such as missing previous life before cancer, their unlived future (Coelho et al., 2020; Pusa et al., 2012). The study conducted by Olson (2014) defined the experiences of carers of a spouse with cancer in survivorship as indefinite loss. It describes a future loss that is possible but not certain. The spouses felt more aware of patient’s and their own mortality, they mourned their lost taken-for-granted future plans (Olson, 2014). Recent studies with survivors showed that death anxiety, disruptions of core beliefs (i.e., we will live together long and die in old age) may enhance fear that cancer will return (Curran et al., 2020; Simonelli et al., 2017). We can assume that spouses who are afraid of losing their wife or husband, who grieve over the disruption of future plans, may experience increased level of fear that cancer will come back. However, larger-scale studies are needed.

When reflecting on the significance of the loss of their spouse, the participants with small children shared that it would be the most unbearable to see their children suffering in the face of their mother’s or father’s death. They accepted the loss of one of the parents in the childhood as the shattering of child’s happiness. There was desire to protect a child from suffering and grief after possible loss. It may put additional tension and helplessness for spouses, as it is impossible to protect others from painful experiences. A sense of insecurity can also be exacerbated by a sense of responsibility for caring for one’s children alone due to the loss of a spouse. So this group of spouses with small kids might be more vulnerable to death-related concerns while experiencing fear of cancer recurrence. Future research should seek to determine, what are the most vulnerable groups of spouses to experience fear of losing the loved one, how experiences of spouses with or without children differ.

Our study showed that the likelihood of recurrence and threat of death evokes the heightened feeling of insecurity in spouses. The sense of insecurity may be related to the risk of losing one’s spouse and that significant relationship. The death of the spouse is associated with loneliness thus insecurity may arise from fear of being left alone. Previous studies revealed that the individual’s sense of security is threatened by the uncertainty and the unpredictability of cancer and lack of control over illness circumstances (Shilling et al., 2017; Strauss et al., 2019). The sense of insecurity may develop into constant state of hypervigilance to external reminders about cancer (Brosschot et al., 2018) and heighten fear of cancer relapse.

Planning for the “worst” scenarios, focus on health behavior helped the spouses to enhance security and control in the face of threat of death. Thewes et al. (2016) study showed that planning can help reduce fear of cancer relapse, feel more in control in face of unknown. On other hand, if you fail to follow everything you planned, it can cause frustration, remorse and greater anxiety. The spouses also used avoidance as escape from the tension provoked by the theme of death: through engagement in active sports activities and increased workload, with the help of laughter and irony, by avoiding any thoughts about recurrence or death. Previous studies found, that survivors with high levels of fear of relapse used more often avoidance as coping strategy (Almeida et al., 2019; Custers et al., 2016; Thewes et al., 2016). Tendency to escape from the situation, retreating from others show positive correlations with the fear of dying and death (Wittkowski, 2016). It is important to mention that in face of an ongoing threat, avoidance can be understood as natural and protective response.

One of important findings of our study is that spouses mentioned not only negative aspects in living with uncertainty and the threat of death. The perception of the limitations of life and its temporality activates engine of life and encourages the spouses to use the time at hand to the fullest, to enjoy life “here and now,” to appreciate life, to focus on positive sides in relationships. A sense of meaning in life, appreciation of life in the face of life constraints and the threat of death can be protective against despair, helplessness, promote a better quality of life, psychological well-being (Breitbart & Poppito, 2014). Further exploration is needed, what are the protective factors against the threat of death and fear of cancer recurrence among cancer survivors’ spouses.

This comprehensive in-depth analysis not only helped to disclose cancer survivors’ spouses experiences of death related worries in fear of cancer recurrence context in detail but also yielded some insights for further research and psychological interventions for spouses. There are several limitations of the study. First of all, this study reflects unique, subjective experience of seven spouses, thus conclusions are limited for this reason in broader cancer survivors’ spouses population. Larger-scale representative studies would allow the validity of these qualitative analysis findings to be verified. It would be useful to disclose similarities and differences of death related concerns in fear of cancer recurrence context among different spouses’ groups (women vs. men, younger vs. elder). Secondly, the analysis was conducted by only one researcher, so a person’s own personality, subjective experience and knowledge could influence findings. In order to increase research credibility and transparency of the researcher’s decisions, interpretations, the researcher participated in individual and group supervisions, wrote diary to reflect research process. Moreover, the study was conducted in Lithuania, one of the Post-Soviet countries, which declared independence 30 years ago. There can be cultural-related factors which influence experiences of spouses: e.g., economical issues that might cause burden for the spouses if the loved one has a great role in maintaining the family, quality of healthcare services available, communication with medical team and involvement of spouses in treatment decision process, avoidance to talk about death and other sensitive topics in society, attitudes and cultural knowledge about cancer. More investigation of death-related concerns underlying fear of cancer recurrence in different cultures is needed. Also future studies should describe the most appropriate methods to help manage spouses‘ the sense of threat of death and worries about cancer relapse.

## Conclusions

Even after the completion of cancer treatment and the achievement of cancer remission, the survivors’ spouses experience the fear of cancer recurrence. They associate cancer recurrence with death and risk of losing their spouse. The threat of death triggers not only the feelings of insecurity, uncertainty about the future and loss of control, but also appreciation of life, focus on positive aspects in relationships with a loved one. Interventions for spouses should include promotion of emotional expression of worst fears, death related concerns underlying fear of relapse. Exposure to their feared outcome in detail, may lessen the threat associated to the thoughts and images of cancer recurrence. By confronting their fears about death, potential loss of the spouse in safe environment, we hope they develop the sense of mastery over uncertainty. Developing new coping strategies (e.g., cognitive reframing, relaxation and mindfulness techniques) to accept and tolerate uncertainty, to lessen insecurity and strengthen control in confrontation with the threat of death may be beneficial.
